# The Relationship Between Laboratory Findings and Mortality in COVID-19 Patients Requiring Intensive Care

**DOI:** 10.7759/cureus.41194

**Published:** 2023-06-30

**Authors:** Ilkay Bahceci, Filiz Mercantepe, Omer Faruk Duran, Soner Yildiz, Kazim Sahin

**Affiliations:** 1 Department of Clinical Microbiology, Faculty of Medicine, Recep Tayyip Erdogan University, Rize, TUR; 2 Department of Internal Medicine, Faculty of Medicine, Recep Tayyip Erdogan University, Rize, TUR

**Keywords:** pandemic, mortality, laboratory parameters, covid-19, biomarker

## Abstract

Introduction: Severe acute respiratory syndrome coronavirus 2 (SARS-CoV-2) shows a wide clinical manifestation from asymptomatic infection to life-threatening respiratory failure. This study aimed to determine the relationship between the survival and demographic data, comorbidity status, and laboratory parameters of coronavirus disease 2019 (COVID-19) patients requiring intensive care.

Material and methods: We retrospectively analyzed 236 patients requiring intensive care whose diagnosis was confirmed by the SARS-CoV-2 reverse transcription-polymerase chain reaction (RT-PCR) test. The patients were divided into two groups in terms of survival. Demographic data; procalcitonin and C-reactive protein (CRP) levels; leukocyte, lymphocyte, and neutrophil counts in hemogram and neutrophil-to-lymphocyte ratio (NLR) levels; and lower respiratory and blood cultures were examined, and the relationships between these parameters and survival were evaluated with hypothesis testing.

Results: In the study, 156 (66.1%) males and 80 (33.9%) females, a total of 236 patients, were included. Sixty-seven (28.3%) surviving patients were determined as Group 1, and 169 (71.7%) deceased patients were determined as Group 2. A statistically significant difference was found between the groups in terms of mean age (p<0.001) and gender distribution (p=0.011). In laboratory parameters, a significant difference was observed between the groups in lymphocyte count (p=0.001), NLR (p<0.001), and procalcitonin levels (p<0.001). Although leukocyte (p=0.075), neutrophil (p=0.031), and CRP (p=0.112) levels were higher in Group 2, there was no statistical difference. Mortality was found to be higher in patients with comorbidity (p=0.012) or co-infection (p=0.002).

Conclusion: High levels of neutrophil count, NLR, and procalcitonin; low lymphocyte count; the presence of comorbidity; and secondary bacterial infection were found to be associated with mortality in COVID-19 patients in the intensive care unit.

## Introduction

Coronavirus disease 2019 (COVID-19) is an infectious disease caused by the severe acute respiratory syndrome coronavirus 2 (SARS-CoV-2), an enveloped and positive polarity ribonucleic acid (RNA) virus in the Coronaviridae family, that emerged in Wuhan, China, at the end of 2019 [1]. Unlike previous coronavirus diseases such as Middle East respiratory syndrome (MERS) and severe acute respiratory syndrome (SARS), it has low pathogenicity and is highly infectious [2]. It has affected 497 million people worldwide and more than 14 million people in Turkey and has caused the death of more than six million people globally [3,4].

COVID-19 often appears with symptoms such as fever, cough, shortness of breath, and loss of taste and smell after the incubation period of 2-14 days and usually transmits with droplets [5]. Although the gold standard method in the diagnosis of the disease is reverse transcription-polymerase chain reaction (RT-PCR), this method has limitations such as low sensitivity and long test duration [6].

SARS-CoV-2 shows a wide clinical diversity from asymptomatic infection to life-threatening respiratory failure [7]. It has been shown that 14%-29% of patients with COVID-19 pneumonia required intensive care [8]. The disease has high mortality in the elderly and people with comorbidities; however, it can also cause severe clinical pictures in young people without comorbidities [9]. To prevent mortality, early detection of patients who require aggressive treatment is very important [10].

COVID-19 is not a localized respiratory tract infection but a multisystemic disease that can cause hyperinflammation and organ failure by triggering inflammatory and immunological responses [11]. Therefore, biomarkers associated with hyperinflammation and organ failure are guides for predicting the clinical picture of the disease [12].

This study aimed to determine the relationship between the survival and demographic data, comorbidity status, and laboratory parameters of COVID-19 patients requiring intensive care.

## Materials and methods

Our study included 236 patients who were followed up in the intensive care unit of Recep Tayyip Erdogan University Training and Research Hospital due to COVID-19 between April 1, 2020, and March 31, 2021, and whose diagnoses were confirmed by the SARS-CoV-2 PCR test. The age, gender, length of stay in the intensive care unit, and comorbidity status of the patients were retrospectively obtained from the hospital information operating system. The hemogram parameters of the patients (neutrophil, leukocyte, lymphocyte, and neutrophil-to-lymphocyte ratio (NLR)), C-reactive protein (CRP), procalcitonin levels, and the presence of secondary bacterial infection were examined. The presence of secondary bacterial infection was evaluated according to blood and lower respiratory tract culture results.

Bacterial culture

The blood cultures of the patients were incubated in the BacT/Alert (bioMérieux, Craponne, France) automated blood culture device in our laboratory. Gram staining is conducted from the growth-detected blood culture bottles and then subcultured to sheep blood agar, eosin methylene blue (EMB) agar, and chocolate agar. Gram staining was performed on the lower respiratory tract samples of the patients. Agar plates were incubated at 35°C-37°C under aerobic conditions. After 24-48 hours of incubation, the colonies were identified with the VITEK 2 compact automated system (bioMérieux, Craponne, France).

Single positive blood culture was considered an etiological agent except for contaminants. Cultures positive for skin flora (e.g., coagulase-negative staphylococci, viridans group streptococci, diphtheroid, *Bacillus* spp., and *Propionibacterium* spp.) were excluded from the study.

In lower respiratory tract samples, a positive culture of possible pathogens was defined as respiratory tract infection. It was accepted that there was no secondary bacterial infection in patients who did not have blood or lower respiratory tract culture requests.

SARS-CoV-2 PCR

Viral nucleic acid extraction was performed from the patients’ combined nasopharyngeal and oropharyngeal swab samples according to the manufacturer’s instructions. Bio-speedy SARS-CoV-2 (2019-nCoV) RT-qPCR Detection Kit (Bioeksen, Istanbul, Turkey) and the Coronex COVID-19 RT-qPCR Detection Kit (DS Bio and Nano Technology, Ankara, Turkey) were used and performed according to the manufacturer’s instructions. Rotor-Gene Q (QIAGEN, Hilden, Germany) and Bio-Rad CFX96 Touch (Bio-Rad Laboratories Inc., Hercules, CA, USA) instruments were used for the tests. Evaluation of the tests was carried out according to the manufacturer’s instructions.

Hemogram parameters and biomarkers

The hemogram parameters of the patients were studied on the Mindray BC-6000 and 6200 (Shenzhen, China) automated hemogram analyzer, and CRP levels were studied on the Beckman Coulter-AU5800 (Brea, CA, USA) automated biochemistry analyzer. Procalcitonin levels were studied with the sandwich time-resolved immunofluorescence (time-resolved fluorescence (TRF)) method in the AQT90 Flex (Radiometer, Copenhagen, Denmark) instrument.

Statistical analysis

All analyses were conducted using the Statistical Package for the Social Sciences (SPSS) version 22.0 (IBM SPSS Statistics, Armonk, NY, USA). Numerical variables were expressed as mean±standard deviation (SD) or median (minimum-maximum) values, and categorical variables were expressed as frequency (number) and percentage (%) values. Normal distribution assumptions were checked using histograms and analytical tests (Kolmogorov-Smirnov/Shapiro-Wilk tests). The Chi-square test was used to compare categorical variables. Since parametric assumptions were not met, numerical variables were analyzed using the Mann-Whitney U test that compares two independent groups. A p-value of <0.05 was considered statistically significant for all data.

For this study, permission was obtained from the Recep Tayyip Erdogan University, Faculty of Medicine, Non-interventional Ethics Committee with decision number 2021/200 dated December 6, 2021, and the Helsinki Declaration criteria were taken into consideration.

## Results

Demographic and laboratory data of 236 patients, 156 (66.1%) male and 80 (33.9%) female, are summarized in Table 1. Patients followed up in the intensive care unit due to COVID-19 were divided into two groups in terms of survival. Sixty-seven (28.3%) surviving patients were determined as Group 1, and 169 (71.7%) deceased patients were determined as Group 2. The mean age in Group 2 was significantly higher than that in Group 1 (p<0.001). It was found that the survival rate was lower in male patients (p=0.011).

**Table 1:** Comparison of COVID-19 Patients by Mortality and Survival COVID-19: coronavirus disease 2019, SD: standard deviation, NLR: neutrophil-to-lymphocyte ratio, CRP: C-reactive protein

	Group 1 (survival) (n=67) (mean±SD)	Group 2 (mortality) (n=169) (mean±SD)	p-value
Age	63±15.3	73.9±10.2	<0.001
Gender (number (%))			
Male	36 (15.2%)	120 (50.8%)	0.011
Female	31 (13.1%)	49 (20.7%)	
Leukocyte (10^3^/uL)	10.2±5	12.9±13.4	0.075
Lymphocyte (10^3^/uL)	0.8±0.4	0.67±0.6	0.001
Neutrophil (10^3^/uL)	9±4.8	11.2±7.6	0.031
NLR	13.7±9.3	23.7±23.8	<0.001
CRP (mg/L)	129.4±96.2	142.4±87.5	0.112
Procalcitonin (ng/mL)	1.7±1.7	3.8±8.8	<0.001
Comorbidity (number (%))	45 (19%)	139 (58.8%)	0.012
Diabetes mellitus (number (%))	21 (8.8%)	61 (25.8%)	0.489
Hypertension (number (%))	38 (16.1%)	116 (49.1%)	0.083
Coronary artery disease (number (%))	13 (5.5%)	50 (21.1%)	0.111
Chronic lung disease (number (%))	5 (2.1%)	27 (11.4%)	0.085
Circulatory system infection (number (%))	4 (1.6%)	40 (16.9%)	0.002
Lower respiratory tract infection (number (%))	7 (2.9%)	51 (21.6%)	0.002

In laboratory parameters, a significant difference was observed between the groups in lymphocyte count (p=0.001), NLR (p<0.001), and procalcitonin (p<0.001) levels. Although leukocyte (p=0.075), neutrophil (p=0.031), and CRP (p=0.112) levels were higher in Group 2, there was no statistical difference. Mortality was found to be higher in patients with comorbidity (p=0.012) or co-infection (p=0.002).

## Discussion

More than six million people around the world and close to 100 thousand people in Turkey lost their lives because of the COVID-19 pandemic. In our study, mortality was detected in 169 (71.7%) of 236 patients followed up in the intensive care unit due to COVID-19. In a meta-analysis conducted in England, the mortality rate in COVID-19 intensive care units was 41.6% [13], while it was found to be 41.2% [14] and 62.59% [15] in studies conducted in our country.

In many studies, the male gender has been associated with high mortality [16]. Similarly, in our study, a statistically significant difference was observed between male and female patients in terms of survival. It is thought that this may be due to the X chromosome, which plays an important role in the immune system and the protective effect of sex hormones against infections in females [17].

An increase in comorbid diseases, weakening of the immune system, and decreased physiological functions are risk factors for COVID-19 in elderly patients [18]. In a study conducted by Teker et al. [19] with 654 patients, they found that the prognosis of the disease worsened, and mortality rates increased with age. In a study conducted in England, it was detected that the risk of mortality was 20 times higher in patients over 80 years compared to the 50-59 age range [20]. In our study, the mean age was found to be significantly higher in deceased patients.

It has been shown in many studies that the disease progresses more severely in the presence of comorbidity, regardless of age [8,10,21]. In our study, the most common comorbidities were diabetes mellitus (DM), hypertension (HT), and coronary artery disease (CAD). While the mortality was higher in patients with comorbidity, there was no significant difference once we looked separately.

CRP is a nonspecific acute phase reactant protein synthesized in the liver, which is frequently used as a diagnostic parameter of infection, associated with the level of inflammation and disease severity [22]. The detection of high CRP levels before the occurrence of tomography findings is effective in the early diagnosis of the disease [12]. In many studies, increased CRP levels have been associated with poor prognosis and mortality [6,18,23]. Contrary to many studies, no statistically significant association was found between mortality and CRP in our study.

Procalcitonin is the pro-peptide of calcitonin secreted from the thyroid gland and is undetectable in a healthy person (<0.1 ng/mL) [24]. The elevation of procalcitonin is associated with systemic bacterial infection and is usually used in differentiating viral and bacterial infections [12]. Continuously elevated procalcitonin levels are indications of bacterial co-infection or serious complications in COVID-19 patients [22]. In a meta-analysis, it was determined that the risk of severe COVID-19 infection increased five times in patients with high procalcitonin levels [6]. Melo et al. [16] found a low correlation between procalcitonin levels and disease severity in their review. In this study, it was observed that high procalcitonin levels had a negative effect on survival.

In addition to biochemical parameters, hematological parameters are also used for evaluations of disease severity and mortality [25]. Complete blood count parameters such as neutrophil, leukocyte, lymphocyte, and NLR can be used in patient follow-up and to determine the treatment approach since they are inexpensive, simple, and reproducible [26].

Lymphopenia is the most common laboratory finding in the early stages of infection among hemogram parameters [27]. As a result of the examination of 28 studies in a meta-analysis, it was determined that the risk of poor prognosis was three times higher in patients with lymphopenia [22].

Neutrophil makes up the majority of leukocytes, and increased neutrophil counts are detected in COVID-19 patients [26]. In a study, it was revealed that the risk of poor prognosis is eight times higher in patients with a high neutrophil count in COVID-19 [28]. The NLR, which is obtained by dividing the number of neutrophils by the number of lymphocytes, is an independent risk factor for the severity of COVID-19 disease [24]. However, its practical use is limited due to the lack of NLR threshold value [29]. The incidence of serious disease in COVID-19 patients aged more than 50 was 50% for patients with NLR ≥ 3.13 [30]. Like previous studies, we found that the neutrophil count and NLR were higher in the deceased group.

In previous influenza pandemics, susceptibility to co-infection and the associated increase in mortality has been detected. That leads to questioning the relationship between survival and secondary bacterial infection in COVID-19 patients [7]. In different studies, the prevalence of secondary bacterial infection varies among patients who died from COVID-19, but it could be up to 50% [7]. In our study, lower respiratory tract and circulatory system infections were examined within secondary bacterial infection, and both were found to be associated with poor prognosis in terms of survival.

Our study has some limitations. Clinical and radiological findings were not included in the study. Only circulatory and lower respiratory tract infections were included in the secondary bacterial infection.

## Conclusions

COVID-19 disease severity classification is important for the appropriate use of resources and the survival of patients who require aggressive treatment. Biochemical and hematological biomarkers are inexpensive, simple, and reproducible tests and should be used in the follow-up of patients and in identifying risk groups. High levels of neutrophil count, NLR, and procalcitonin; low lymphocyte count; the presence of comorbidity; and secondary bacterial infection were found to be associated with mortality in COVID-19 patients in the intensive care unit.

## References

[1] Tabassum T, Rahman A, Araf Y, Ullah MA, Hosen MJ (2021). Prospective selected biomarkers in COVID-19 diagnosis and treatment. Biomark Med.

[2] Das B, Bhatia SY, Pal PM (2021). Evaluation of the role of routine laboratory biomarkers in COVID-19 patients: perspective from a tertiary care hospital in India. Indian J Clin Biochem.

[3] Balcı A, Yıldız E, Selendili O (2022). TR Ministry of Health COVID-19 information platform. J Contemp medıcıne.

[4] (2022). World Health Organization: Coronavirus (COVİD-19) dashboard. https://covid19.who.int/.

[5] Balci A, Yildiz E, Selendili O (2021). New markers in the differentiation of Covid 19 disease and pneumonia: neutrophil and platelet lymphocyte ratio. J Contemp Med.

[6] Suklan J, Cheaveau J, Hill S (2021). Utility of routine laboratory biomarkers to detect COVID-19: a systematic review and meta-analysis. Viruses.

[7] Bahceci I, Yildiz IE, Duran OF, Soztanaci US, Kirdi Harbawi Z, Senol FF, Demiral G (2022). Secondary bacterial infection rates among patients with COVID-19. Cureus.

[8] Sabaz MS, Özdemir F, Özakin O, Türkmen ÜAygen, Dinç V (2021). Intensive care unit admission parameters for patients with COVID-19. Med J Bakirkoy.

[9] Şahin L, Gür A (2021). The relationship between procalcitonin, D-dimer, ferritin, troponin and lactate levels with COVID-19. Acta Medica Alanya.

[10] Filiz M, Yilmaz G, Fidan G (2021). A tertiary care hospital experience during the COVID-19 pandemic. Flora J Infect Dis Clin Microbiol.

[11] Samprathi M, Jayashree M (2020). Biomarkers in COVID-19: an up-to-date review. Front Pediatr.

[12] Talan L (2021). CRP/procalcitonin as a biomarker during the COVID-19. Use of biomarkers in clinical practice in the COVID-19 process.

[13] Armstrong RA, Kane AD, Cook TM (2020). Outcomes from intensive care in patients with COVID-19: a systematic review and meta-analysis of observational studies. Anaesthesia.

[14] Kosovalı BD, Yurduseven Civgin E, Ozkan E, Tunçer Peker T, Mutlu M (2022). Clinical severity and mortality predictors in COVID-19 intensive care patients: CTSS and CO-RADS. Turkish J Clin Lab.

[15] Kavutlu M, Engin E, Senol D (2022). Effect of prone position on treatment course in COVID-19 patients. Turk J Intens Care.

[16] Melo AK, Milby KM, Caparroz AL (2021). Biomarkers of cytokine storm as red flags for severe and fatal COVID-19 cases: a living systematic review and meta-analysis. PLoS One.

[17] Cattelan AM, Di Meco E, Trevenzoli M (2020). Clinical characteristics and laboratory biomarkers changes in COVID-19 patients requiring or not intensive or sub-intensive care: a comparative study. BMC Infect Dis.

[18] Unver-Ulusoy T, Demirkose M, Bilek H (2021). Diagnostic utility and prognostic value of basic laboratory parameters in COVID-19 (Article in Turkish). Klimik J.

[19] Teker AG, Emecen AN, Girgin S (2021). Epidemiological characteristics of covid-19 cases in a university hospital in Turkey (Article in Turkish). Klimik J.

[20] Kazancioglu L, Erdivanli B, Kazdal H (2021). Effectiveness of laboratory parameters as morbidity and mortality indicators in patients with coronavirus disease-2019 admitted to the intensive care unit. Turkish J Intensive Care.

[21] Tjendra Y, Al Mana AF, Espejo AP, Akgun Y, Millan NC, Gomez-Fernandez C, Cray C (2020). Predicting disease severity and outcome in COVID-19 patients: a review of multiple biomarkers. Arch Pathol Lab Med.

[22] Malik P, Patel U, Mehta D (2021). Biomarkers and outcomes of COVID-19 hospitalisations: systematic review and meta-analysis. BMJ Evid Based Med.

[23] Peiró ÓM, Carrasquer A, Sánchez-Gimenez R (2021). Biomarkers and short-term prognosis in COVID-19. Biomarkers.

[24] Ponti G, Maccaferri M, Ruini C, Tomasi A, Ozben T (2020). Biomarkers associated with COVID-19 disease progression. Crit Rev Clin Lab Sci.

[25] Gursoy Doruk O, Ormen M, Tuncel P (2021). Biochemical and hematological parameters in COVID-19 (Article in Turkish). J DEU Med.

[26] Yildirim O, Bayram M, Ozmen RS (2021). Evaluation of hematological indices in terms of COVID-19 related mortality and ICU admission. J Health Sci Medicine.

[27] Ciaccio M, Agnello L (2020). Biochemical biomarkers alterations in coronavirus disease 2019 (COVID-19). Diagnosis (Berl).

[28] Henry B, Cheruiyot I, Vikse J (2020). Lymphopenia and neutrophilia at admission predicts severity and mortality in patients with COVID-19: a meta-analysis. Acta Biomed.

[29] Israni A, Goulden CJ, Harky A (2021). Laboratory biomarkers and prognosis in Covid-19, where do we stand?. Rev Med Virol.

[30] Pourbagheri-Sigaroodi A, Bashash D, Fateh F, Abolghasemi H (2020). Laboratory findings in COVID-19 diagnosis and prognosis. Clin Chim Acta.

